# Confidentiality at the interface of an adolescent psychotherapy service

**DOI:** 10.1192/bjo.2021.599

**Published:** 2021-06-18

**Authors:** Rhianne Thomas, Lawrence Congdon, Sheva Habel

**Affiliations:** Tavistock and Portman NHS Foundation Trust

## Abstract

**Aims:**

Aims included to explore how, within a London trust, staff at the interface between patients, relatives and access to services view their understanding of confidentiality, and to determine ways to improve knowledge if needed.

**Background:**

Confidentiality is essential to the trust and development of clinician-patient relationships. National policies set guidance on how confidential information should be recorded, secured and shared. However, confidentiality breaches are reportedly common within health professions. Working with adolescent patient groups brings additional issues regarding confidentiality. Care-givers who contact services, often desiring containment, may experience a sense of uncertainty when confidentiality policy prevents details being shared about a young person's clinical experience.

**Method:**

Stakeholders were identified from the multidisciplinary team, with a collaborative rather than ‘top-down’ approach. Administrators in patient-facing roles were surveyed to ascertain current understanding and frequency of involvement in confidentiality issues. Based on feedback, a flowchart prompt was designed, ensuring it reflected best practice. Qualitative and quantitive data were collected before and after a two month implementation period.

**Result:**

All respondents (n = 10) dealt with confidentiality issues at work, with 50% experiencing issues daily. 33% respondents did not feel confident dealing with confidentiality queries at work. The majority (60%) had received confidentiality training, but all respondents thought extra information would be useful. Of possible interventions, 70% supported a flowchart. Following an implementation period, 100% respondents re-surveyed agreed they felt confident dealing with issues related to confidentiality at work. The majority of respondents had used the flowchart and found it useful (83%). Qualitative data gathered suggested rolling-out the project elsewhere.

**Conclusion:**

A lack of confidence surrounding issues with confidentiality, including information sharing, was identified. This can negatively impact patient engagement and delivery of care. The introduction of the confidentiality flowchart demonstrated improved understanding of, and confidence in, patient confidentiality issues. The small sample size means there are limitations in extrapolating findings to wider contexts. However, it is likely that more confidentiality training and practical information for NHS staff at the interface between patients, clinicians and services would reduce the risk of confidentiality breaches and reinforce positive relationships with services.

